# Optimized MaxEnt modeling predicts the distribution change of *Chaenomeles* sp*eciosa* (Sweet) Nakai in China under global climate change

**DOI:** 10.3389/fpls.2026.1737731

**Published:** 2026-02-06

**Authors:** Hongjian Shen, Shasha Sun, Yuxue Cheng, Emelda Rosseleena Rohani, Qingying Fang, Rongchun Han, Xiaohui Tong

**Affiliations:** 1 School of Pharmacy, Anhui University of Chinese Medicine, Hefei, China; 2 Institute of Systems Biology, Universiti Kebangsaan Malaysia, Bangi, Malaysia; 3 AHUCM-UKM Joint Laboratory for Traditional Medicine Quality Standardization, Anhui University of Chinese Medicine, Hefei, China; 4 School of Life Sciences, Anhui University of Chinese Medicine, Hefei, China

**Keywords:** ArcGIS, *Chaenomeles speciosa*, climate change, CMIP6, MaxEnt, potential distribution, species distribution modeling

## Abstract

Climate change is influencing the distribution of medicinal plants, necessitating the need for the development of precise models to predict habitat changes. However, studies on the habitat dynamics of *Chaenomeles* sp*eciosa*, an important medicinal herb, under current and future climate scenarios are lacking. In this study, we applied an optimized maximum entropy algorithm integrated with ArcGIS, and 157 occurrence points of *C.* sp*eciosa* along with 10 environmental variables to predict its potentially suitable distribution under both current and future climate scenarios (SSP245 and SSP585). The model performed well with an average area under the curve (AUC) of 0.908 and a true skill statistic (TSS) of 0.674. The key factors were Bio_14 (Driest Month), Bio_4 (Temperature Seasonality), elevation, and Srad_10 (October solar radiation). Currently, the species has an estimated total potential distribution range of approximately 328.40 × 10^4^ km^2^, and the most suitable habitats are primarily located in central and eastern China. Projections indicate that under future climate scenarios, although the total suitable region increases, the proportion of high-suitability regions notably declines. Core regions are expected to contract as peripheral regions expand, and the distribution centroid will shift nonlinearly within Hubei Province. Therefore, we suggest prioritizing the monitoring of the spatial redistribution of suitable habitats for the future conservation and sustainable use of *C.* sp*eciosa*.

## 1 Introduction

The global distribution of plant species is undergoing considerable reorganization, predominantly as a result of the widespread effects of climate change (Payne and Bro-Jørgensen, 2016). Altering critical environmental factors, such as temperature and precipitation, directly constrain plant growth and trigger the reconfiguration of their geographical distribution (Pecl et al., 2017). Plants migrate to higher altitudes and latitudes in pursuit of suitable climatic niches. A comprehensive global assessment of mountain plants indicates that species are currently migrating upwards at an average rate of approximately 11 m per decade (Steinbauer et al., 2018); concurrently, temperate tree species are exhibiting pronounced northward expansion (Li et al., 2013), plant migration responses are highly variable, with their direction and rate influenced by intricate interactions between local water-heat combinations, interspecies interactions, and abiotic factors (Lenoir et al., 2020). For example, in regions experiencing increased water stress, some species exhibit anomalous migration patterns towards lower altitudes, highlighting the pivotal role of altered precipitation patterns (Crimmins et al., 2011). Satellite observations of North American vegetation have provided additional evidence that climate change is actively influencing large-scale shifts in forest community composition, with the distribution ranges of warm-adapted species continuously expanding (Rogers et al., 2018). Understanding these complex and variable response patterns is critical to predict biodiversity trajectories, assess ecosystem services, and formulate effective conservation strategies.

Species distribution models (SDMs) are vital tools in ecology and conservation biology for predicting the potential geographical distribution of species. Specifically, the maximum entropy (MaxEnt) model, which is based on the principle of maximum entropy, is preferred for its exceptional performance (Harte and Newman, 2014; Wang et al., 2024). Compared to traditional models, such as Climex (Souza et al., 2023) and GARP (Yang et al., 2020), MaxEnt demonstrates notable advantages in handling complex nonlinear relationships, utilizing small sample sizes, and effectively correcting for sampling bias, thereby typically achieving higher predictive accuracy (Shao et al., 2023). However, despite its user-friendly default parameter settings for novices, this “black-box” approach has inherent risks of excessive model complexity and overfitting, potentially compromising the ecological interpretation and transferability of predictions. Consequently, current best practices strongly recommend parameter optimization before model construction. This involves screening feature combinations and regularization multipliers using tools such as ENMeval in R to determine optimal configurations (Luo et al., 2025). This optimized MaxEnt modeling procedure has been extensively and successfully applied across multiple cutting-edge domains, including prioritizing conservation regions for endangered species and assessing the risk of spreading invasive alien species. This study provides a robust scientific foundation for biodiversity conservation and ecosystem management (Elith and Leathwick, 2009).

*Chaenomeles* sp*eciosa* (Sweet) Nakai, a deciduous shrub belonging to the genus *Chaenomeles* within the Rosaceae family, is extensively cultivated in Chinese provincial-level administrative regions, such as Chongqing, Anhui, and Hubei. This species is important for both medicinal and food uses (Huang et al., 2018). The dried, near-ripe fruit, referred to as “Mugua” in China, is traditionally used for its effects in relaxing tendons, activating meridians, harmonizing the gastrointestinal function, and alleviating dampness. Clinically, it is frequently used to treat rheumatic arthralgia, tendon-meridian constriction, vomiting, and diarrhea with muscle cramps (Xu et al., 2023; Zhang et al., 2010). Its primary active constituents include triterpenoids, phenolics, flavonoids, and polysaccharides, which exhibit strong anti-inflammatory, antioxidant, and immune regulatory effects (Pei et al., 2025; Marat et al., 2022; Hou et al., 2025). Sustained growth in market demand has placed considerable pressure on wild resources, and current cultivation techniques continue to encounter challenges, such as germplasm degradation and pest and disease control (Groenewald et al., 2024). Climate change has profoundly influenced the geographical distribution patterns of medicinal plants through alterations in temperature and precipitation factors (Ren et al., 2023; Zhang et al., 2023). However, studies on the environmental adaptation of *C.* sp*eciosa* to projected climate shifts and the evolution of suitable distribution regions remain limited. To address this gap, this study employed an optimized MaxEnt model and ArcGIS to systematically evaluate the distribution patterns of potentially suitable habitats for *C.* sp*eciosa* under current climatic conditions and two climatic change scenarios (SSP245 and SSP585), specifically for the 2050s (2041–2060) and the 2070s (2061–2080). By interpreting the model-derived outputs, including the variable rankings and response curves, we identified the dominant climatic drivers that influenced their distribution. We comprehensively analyzed the ecological threshold ranges of these factors, thereby defining the climatic limits of *C.* sp*eciosa* under climate change.

This study aimed to integrate the optimized MaxEnt model with ArcGIS to (i) simulate and delineate suitable habitat classes for *C.* sp*eciosa* under current climatic conditions, (ii) identify and quantify key environmental drivers and their ecological thresholds influencing its distribution pattern, and (iii) project temporal and spatial trends in suitable distribution regions under various climate scenarios (SSP245 and SSP585) for the 2050s and the 2070s.

## 2 Materials and methods

**Figure 1:**
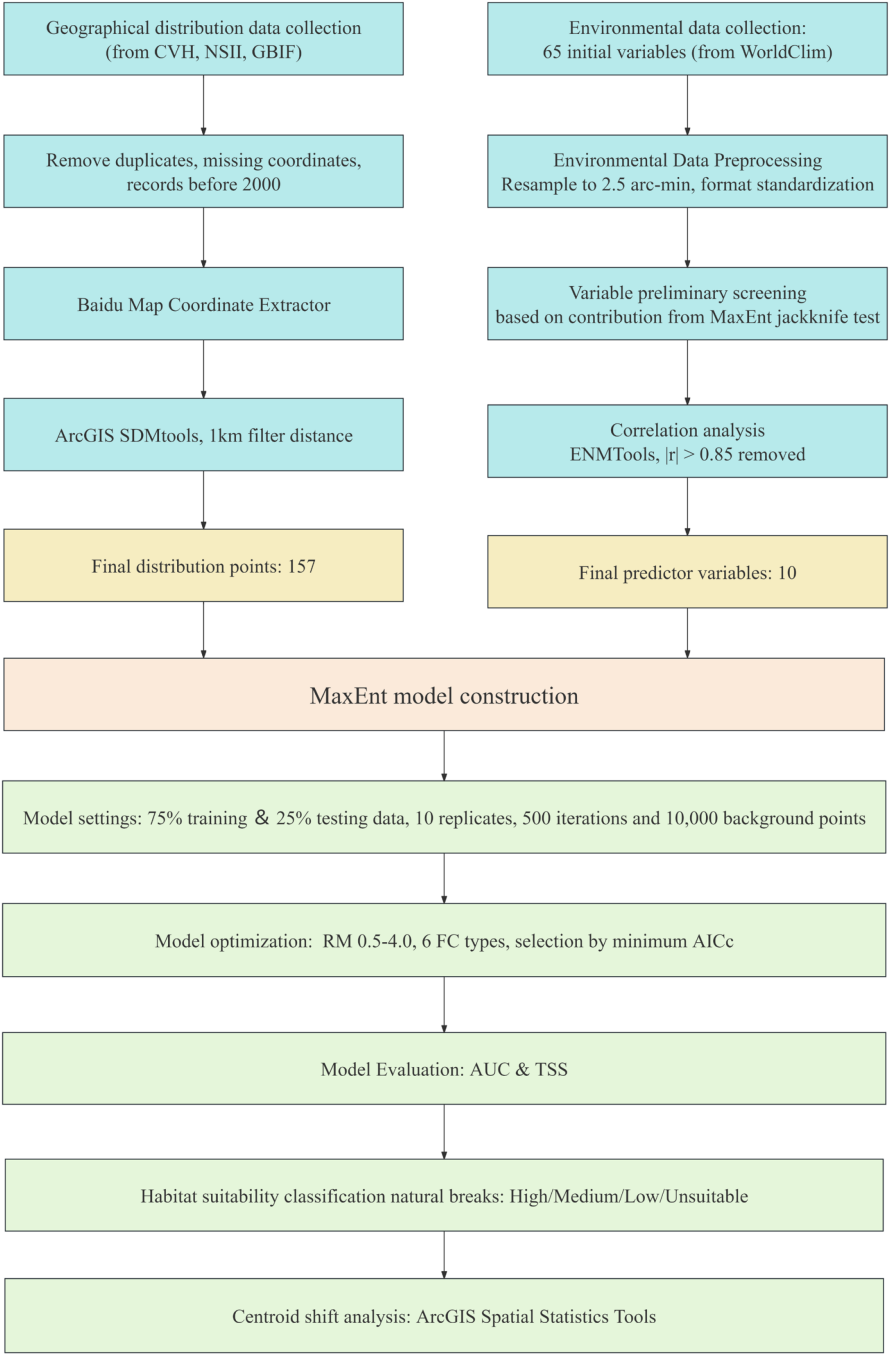
Methodological workflow of the study.

The methodological workflow of this study is illustrated in **Figure 1**. It comprises four sequential phases: (i) data collection and preparation, (ii) environmental variable screening, (iii) MaxEnt model development and optimization, and (iv) spatial analysis and future projection. Each phase is described in detail in the following subsections.

### 2.1 Natural distribution records and data processing of *C.* sp*eciosa*

**Figure 2:**
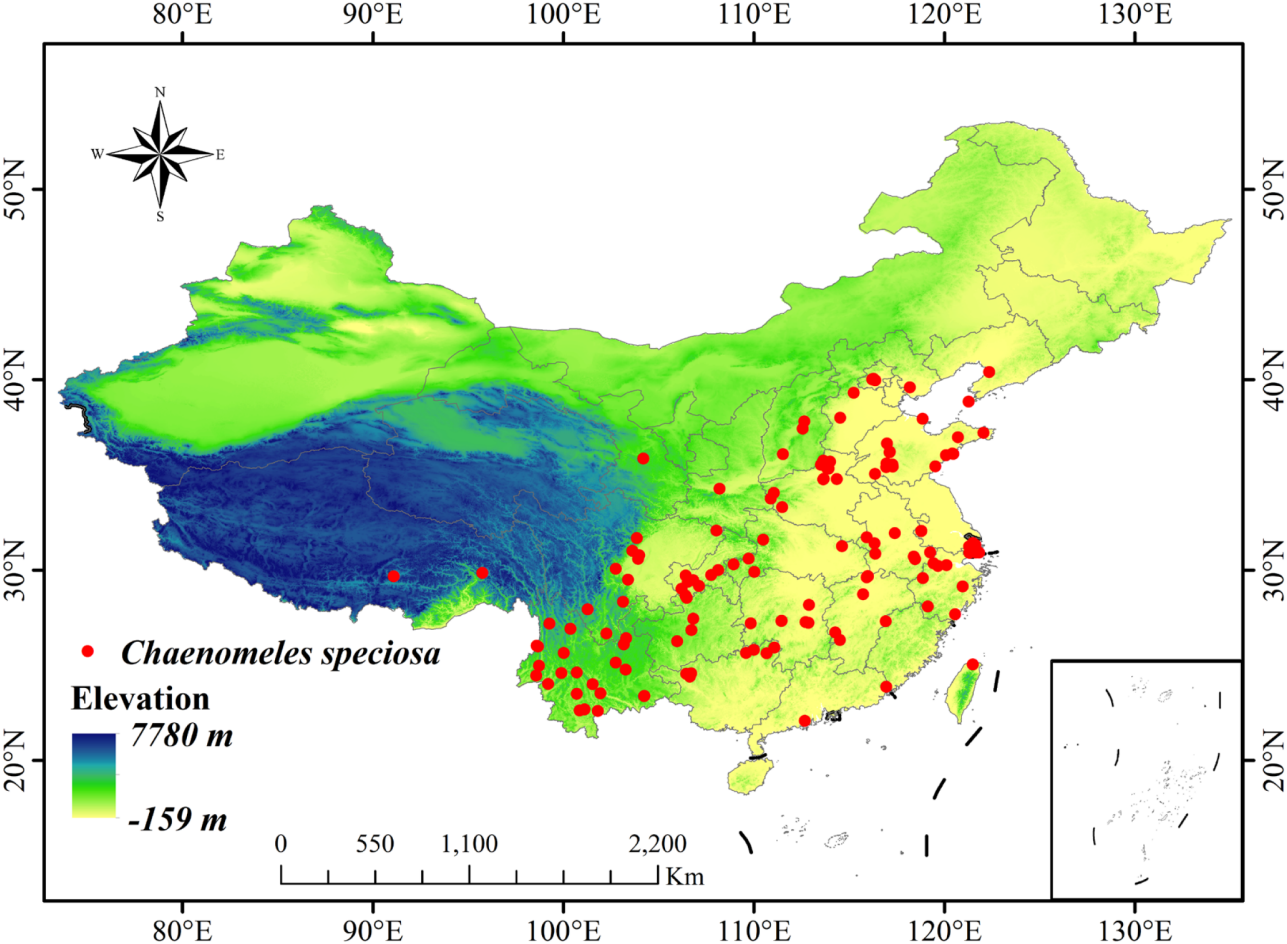
Distribution records of *Chaenomeles* sp*eciosa* in China.

Geographical distribution data for *C.* sp*eciosa* were sourced from the China Virtual Herbarium (CVH, http://www.cvh.ac.cn/), the National Specimen Information Infrastructure (NSII, http://www.nsii.org.cn), and the Global Biodiversity Information Facility (GBIF, https://www.gbif.org). After screening the raw data, duplicate records, specimens lacking precise geographic locations, and specimens collected before 2000 were excluded. The Baidu Map Coordinate Extractor (https://lbs.baidu.com/maptool/getpoint) was used to supplement the latitude and longitude coordinates of specimens with missing coordinates, resulting in 163 initial distribution records. A significant level of spatial autocorrelation is present in environmental variables over short ranges (Dormann et al., 2007). To mitigate potential bias from spatial autocorrelation in species distribution modeling, the SDMTools package was employed within ArcGIS 10.8 to apply spatial thinning with a 1 km filter distance. This ensured that only the most representative single distribution record was retained per 1 km × 1 km grid cell (Wu et al., 2025). Following this process, the final dataset comprised 157 valid distribution points, which were used for all subsequent modeling and analysis (**Figure 2**).

### 2.2 Environmental data sources and preprocessing procedures

Climate data were obtained for the contemporary period (1970–2000) and two future periods (the 2050s and 2070s). Owing to the pronounced time lag in the response of ecosystems to climate change, the 2050s and 2070s were selected as representative periods for the future scenarios. This approach enables the separate assessment of species distribution patterns, indicating both the near-term response to climate change and the long-term potential for habitat loss or shifts in adaptability thresholds owing to cumulative effects (Fu et al., 2024). Nineteen bioclimatic variables together with 12-monthly data on solar radiation, vapor pressure, and wind speed were obtained from the WorldClim database (https://worldclim.org/). Simultaneously, the extraction of topographic variables from the platform’s elevation data produced three key factors: elevation, slope, and aspect. Soil environmental data were sourced from the World Soil Database (HWSD, http://www.fao.org/soils-portal/soil-survey/soil-maps-and-databases/harmonized-world-soil-database-v12/en/), with selected variables including soil effective water content (AWC_CLASS), topsoil textural class (T_TEXTURE), soil drainage class (DRAINAGE), topsoil gravel content (T_GRAVEL), topsoil reference bulk density (T_REF_BULK), topsoil organic carbon content (T_OC), and pH (T_PH_H2O). All environmental layers underwent uniform spatial clipping, resolution standardization (2.5 arc-min), and format conversion using SDMTools. Bioclimatic data for future periods were sourced from the WorldClim platform, which integrates the subscale data from the BCC-CSM2-MR climate model within the Sixth Coupled Model Intercomparison Project (CMIP6). The BCC-CSM2-MR model employed in this study demonstrates good applicability to climate simulations in China (O’Neill et al., 2017). An analysis of the potential changes in suitable regions for *C.* sp*eciosa* under two representative Shared Socioeconomic Pathways (SSP245 and SSP585) was conducted (Daï et al., 2023). All environmental layers were uniformly processed to a 2.5-arc-min resolution, with format standardization and spatial cropping performed using SDMTools.

**Figure 3:**
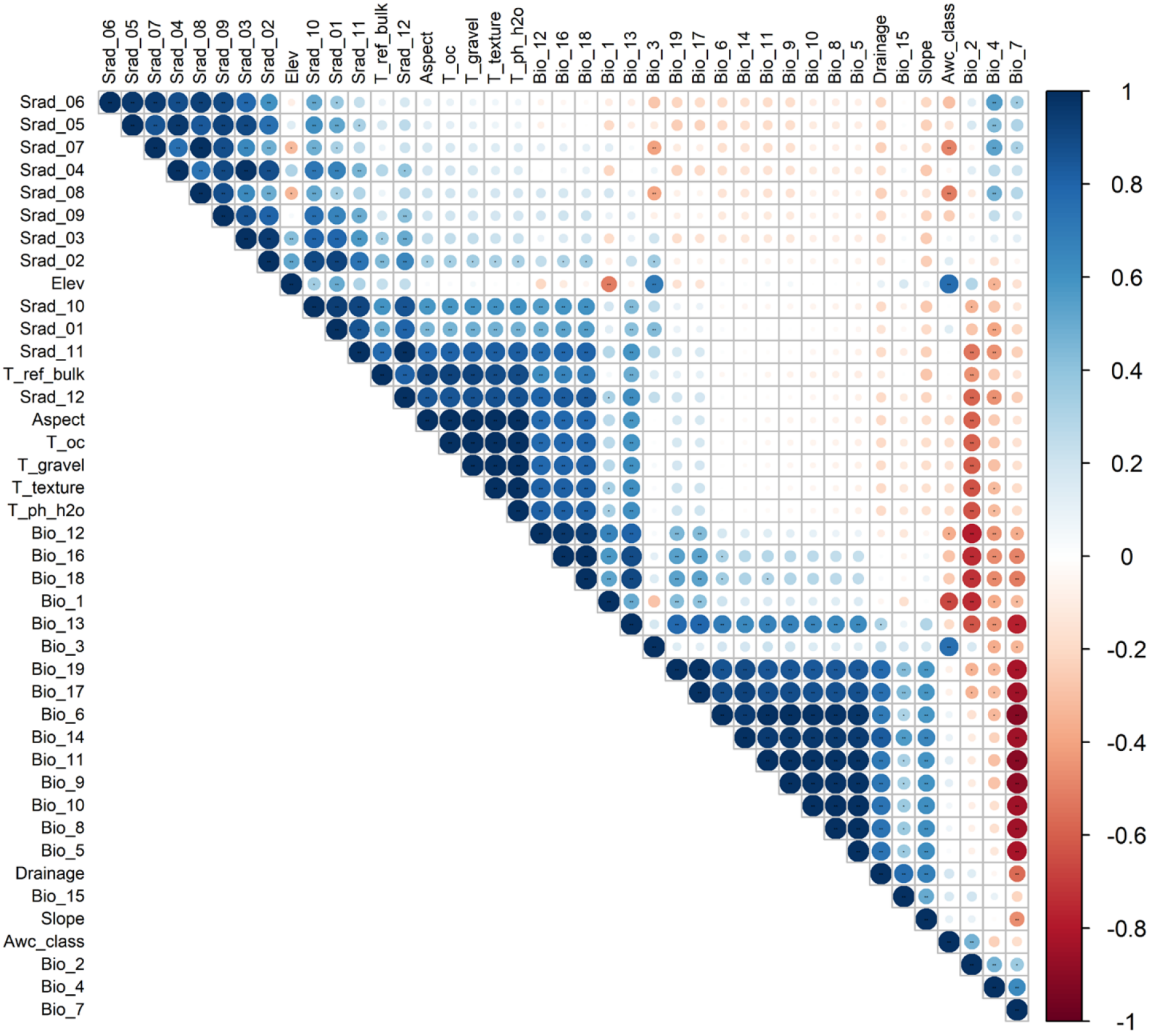
Environmental factors correlation analysis heatmap.

Based on 65 environmental factors (**Supplementary Material 1**), a MaxEnt model was constructed for trial runs. To mitigate potential overfitting resulting from high correlations among environmental variables (Huang et al., 2024), we initially assessed each factor’s contribution to model predictions using the jackknife test in MaxEnt, and subsequently excluded factors exhibiting low contribution rates. For the remaining 41 factors, correlation analysis was performed using ENMTools (**Figure 3**), with the Pearson correlation coefficient threshold set at 0.85. Environmental factors (|r| > 0.85) were considered to exhibit strong collinearity (Zhao et al., 2024). These factors were merged and filtered based on the Jackknife test results, with selection priority given to those with higher contribution rates. After completing this screening procedure, a collection of 10 environmental factors was obtained, which were utilized in the construction of the species distribution model. (**Table 1**). This procedure effectively mitigates the risk of model overfitting while ensuring the ecological significance of key environmental drivers (Meyerhenke et al., 2018).

**Table 1:** Environmental factors ultimately used for modeling.

Environmental factor	Description	Unit
Bio_3	Isothermality (BIO2/BIO7) (× 100)	°C
Bio_4	Temperature Seasonality (Standard deviation × 100)	°C
Bio_14	Precipitation of Driest Month	mm
Bio_15	Precipitation Seasonality (Coefficient of variation)	mm
Elevation	Altitude	m
Aspect	Orientation of the slope	—
Slope	Gradient of the terrain	°
Srad_08	August solar radiation	KJ/m^2^/day
Srad_10	October solar radiation	KJ/m^2^/day
Awc_class	Soil-effective water content	%

### 2.3 Species distribution models

To simulate potentially suitable distribution regions for *C.* sp*eciosa*, we imported its distribution records, along with filtered environmental variables, into the MaxEnt model (version 3.4.4). Using a Logistic output format to evaluate model performance, we implemented a random split of the occurrence records; 75% was the training set for model development. The remaining 25% comprised the validation test set (Dong et al., 2022). To enhance the robustness and generalizability of our predictions, a bootstrap procedure with 10 replicates was used (Ouyang et al., 2022). An iteration ceiling of 500 was established as a safeguard against model overfitting, and the environmental context of the study area was characterized by generating 10,000 background points. When species presence data are limited, using a larger set of background points can help mitigate possible sampling bias. The predictive accuracy of our model was quantified by analyzing the receiver operating characteristic (ROC) curve and calculating the area under the curve (AUC). The AUC values range from 0 to 1, with higher values indicating stronger correlations between the predicted species distributions and environmental factors, indicating superior model performance. The AUC accuracy grading criteria employed in this study were as follows: 0.50–0.60 (failing), 0.60–0.70 (poor), 0.70–0.80 (fair), 0.80–0.90 (good), and 0.90–1.00 (excellent) (Chen et al., 2025). Furthermore, we employed the Jackknife test to quantify the independent contribution of each environmental variable to model construction, thereby identifying the key ecological factors influencing the distribution of *C.* sp*eciosa*.

To address the potential limitations of the AUC metric in specific contexts, we introduced the true specific sensitivity (TSS) statistic. By integrating model sensitivity and specificity, the TSS effectively mitigates sample imbalance bias in species distribution data, resulting in a more balanced evaluation (Allouche et al., 2006). TSS values range from –1 to 1, with values approaching 1 indicating better model performance. The TSS value between 0.6 and 1 indicates good predictive capability. All TSS computations in this study were conducted using the R software (version 4.5.1, R Foundation for Statistical Computing, Vienna, Austria).

### 2.4 Systematic parameter tuning for MaxEnt modeling

The regularization level of MaxEnt is primarily determined by two parameters: the regularization multiplier (RM) and feature combinations (FC) (Yan et al., 2021). The model may employ five fundamental feature functions: linear (L), quadratic (Q), hinge (H), product (P), and threshold (T). This study employed a systematic experimental design to identify the optimal parameter combinations. The regularization multiplier was set incrementally over the range 0.5–4.0, with increments of 0.5. Six distinct feature-class combinations (L, H, LQ, LQH, LQHP, and LQHPT) were evaluated using a cross-validation framework. For model fit assessment, the Akaike Information Criterion Correction (AICc) metric was employed as the evaluation criterion (Muscarella et al., 2014; Wu et al., 2025). The specific computational process was implemented using the ENMeval package in the R programming platform, resulting in AICc quantification values for the maximum entropy model under various parameter configurations. During model selection, the AICc metric was used to assess the relative fit of the candidate models to the optimal model. This value was calculated by subtracting the minimum AICc value of the candidate models from the AICc value of each model. When ΔAICc is <2, the model is considered to have significant statistical support; if ΔAICc exceeds 10, the model’s credibility is considered significantly insufficient (Olofsen and Dahan, 2013). In this study, the optimal modeling scheme with ΔAICc=0 was ultimately selected for simulating *C.* sp*eciosa* habitat.

### 2.5 Data preparation methodology

To visually represent the potential habitat suitability regions for *C.* sp*eciosa*, the model-predicted average species occurrence probabilities were imported into ArcGIS for spatial mapping and classification. Natural breakpoint classification was employed to segment the suitability probabilities. This method determines classification intervals based on the inherent natural groupings of the data, thereby minimizing intra-class variation. Ultimately, four suitability classes were generated: high (0.5–1.0), medium (0.3–0.5), low (0.1–0.3), and unsuitable (0–0.1). This classification scheme, which partitions the continuous probability of occurrence (0–1) into distinct ecological suitability levels, is a well-established practice in species distribution modeling (Zhang et al., 2022). Widely adopted for area calculation and spatial planning, these thresholds are tied to interpretable gradients in habitat suitability. Compared to merely analyzing fluctuations in the area of each suitability class, the migration trajectory of their distribution centers more profoundly reveals the temporal restructuring process of the spatial pattern of suitable regions (Kong et al., 2019). Based on this, the present study quantified areas with different suitability grades across periods. Furthermore, using ArcGIS spatial statistics tools, the present study transformed the continuous suitable distribution surface of *C.* sp*eciosa* into representative distribution centers (Cong et al., 2020; Ahmadi et al., 2023) to characterize the spatial aggregation centers and dynamic shifts of its overall suitable habitat.

## 3 Results

### 3.1 Optimization and predictive performance evaluation

**Figure 4:**
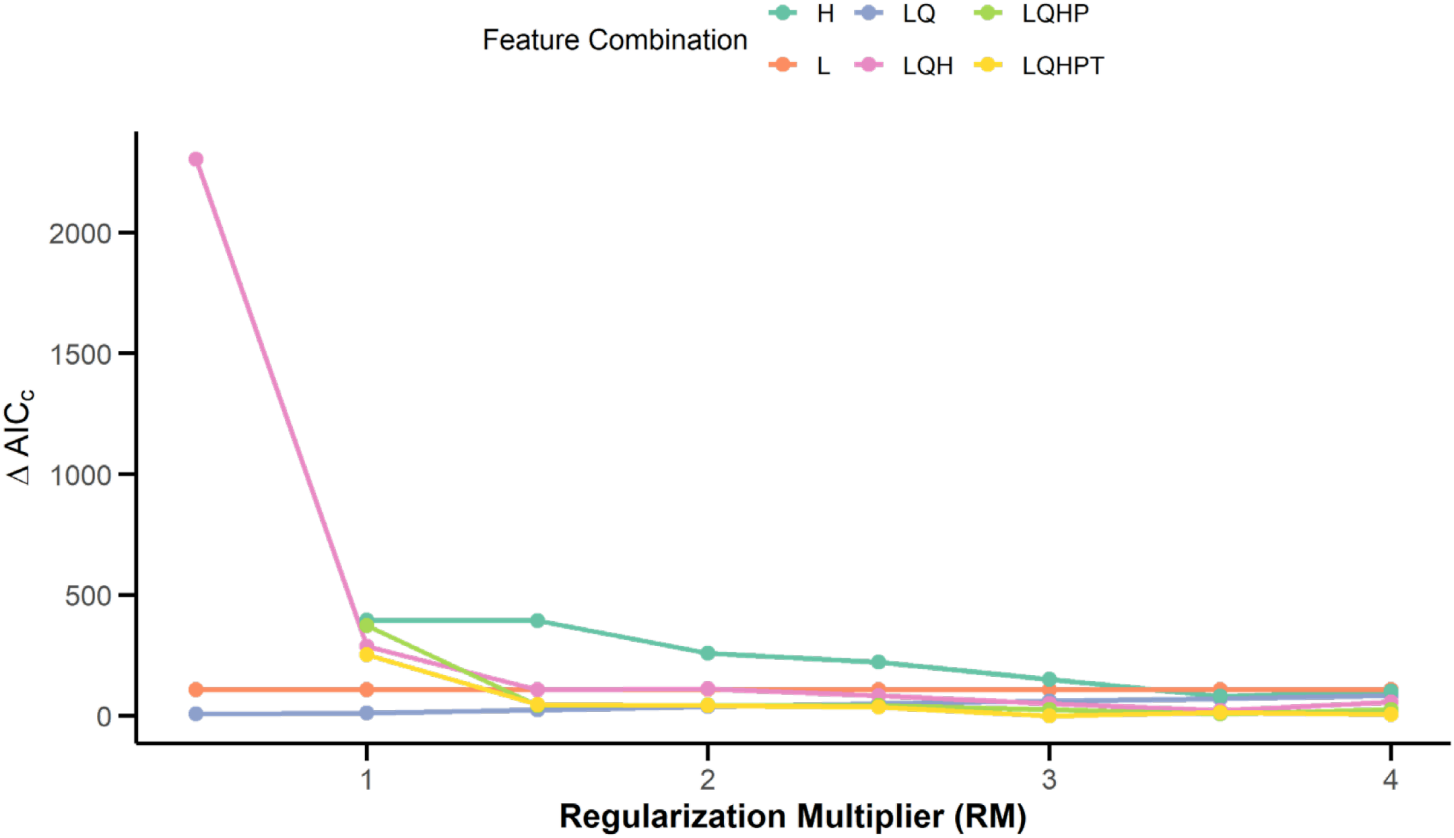
Parameter optimization results.

**Figure 5:**
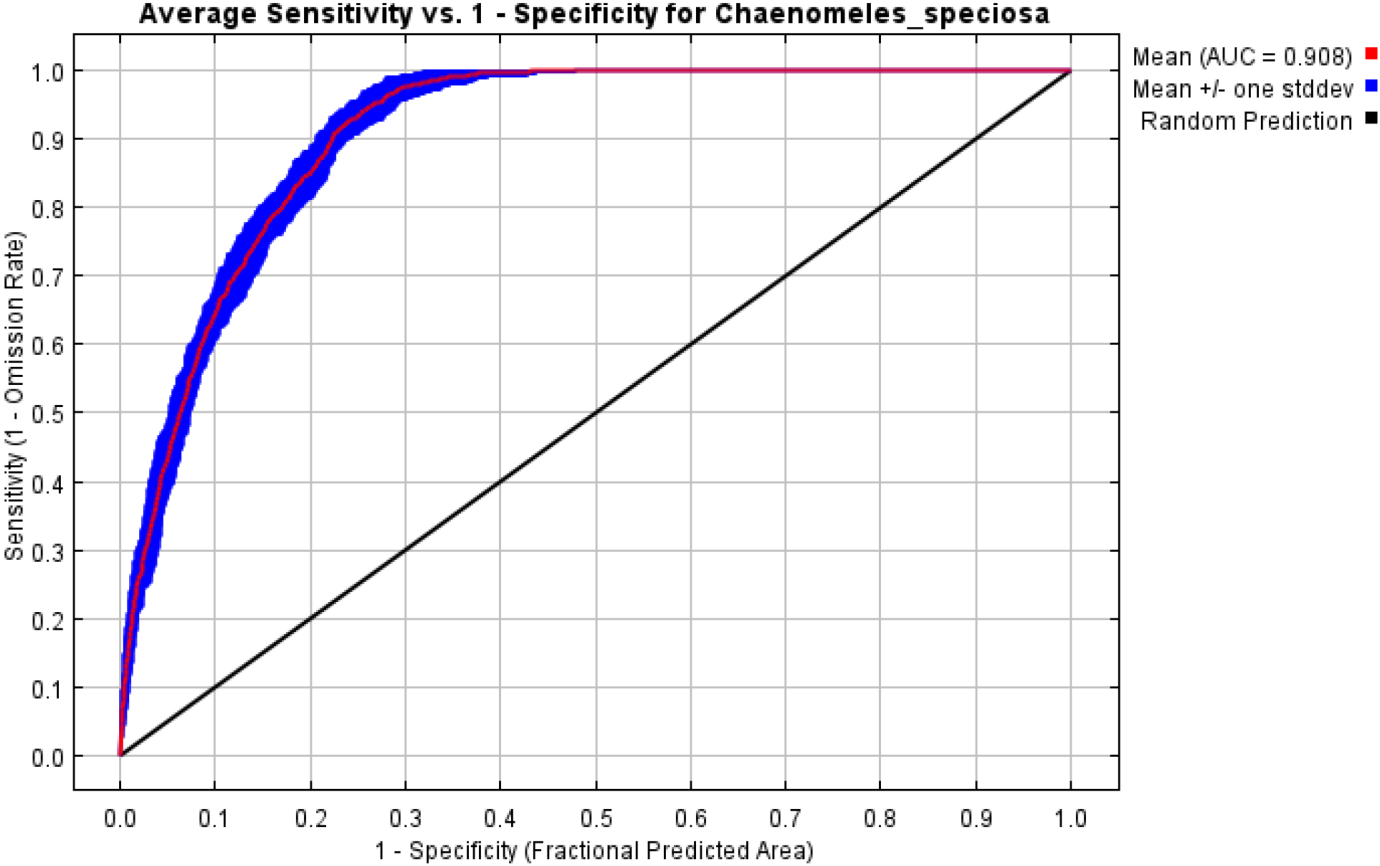
Response of the ROC curve under the MaxEnt model for *C.* sp*eciosa*.

Initial modeling using default parameters (RM = 1, FC = LQH) resulted in a ΔAICC of 287.87, indicating a risk of overfitting. To optimize model performance, parameters were systematically tuned using ENMeval in R. Setting RM = 3 and FC = LQHPT reduced ΔAICC to 0, indicating this configuration as the optimal model setting (**Figure 4**). With these optimized parameters, the predictive performance of the model significantly improved, achieving an average AUC of 0.908 (**Figure 5**) and a mean TSS of 0.674. Both evaluation metrics confirmed the excellent predictive capability and robustness of the proposed model.

### 3.2 Environmental drivers of *C.* sp*eciosa* distribution patterns

**Figure 6:**
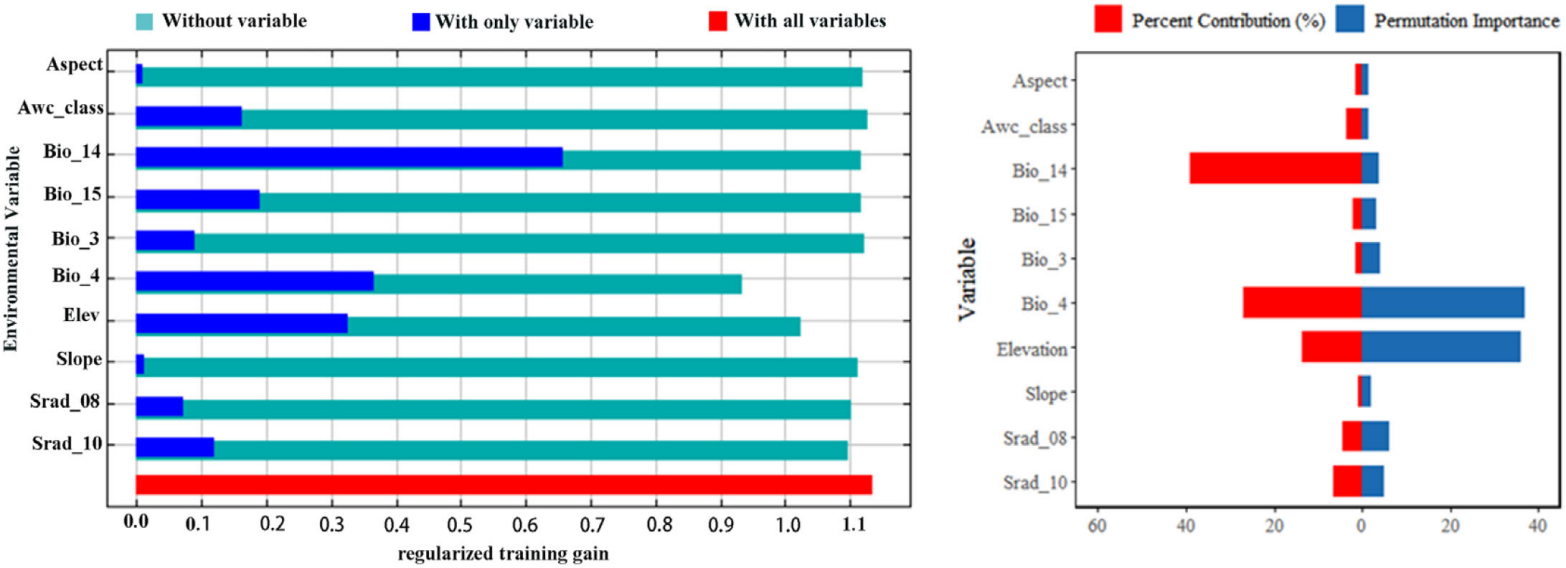
Jackknife test of environmental variables and contributions.

The model results (**Supplementary Material 2**) indicated that Precipitation of Driest Month (Bio_14), Temperature Seasonality (Bio_4), elevation, and October solar radiation (Srad_10) collectively explained 86.3% of the variation in habitat suitability for this species. Specifically, the Precipitation of Driest Month was identified as the most explanatory environmental factor, accounting for 39.1% of the contribution. The jackknife test further confirmed that Temperature Seasonality (36.9%) and elevation (36.2%) had the highest replacement importance in the model. A comprehensive analysis indicated that moisture conditions, temperature range, and solar radiation energy collectively constituted the dominant environmental influences that determined the geographical distribution patterns of this species (**Figure 6**).

**Figure 7:**
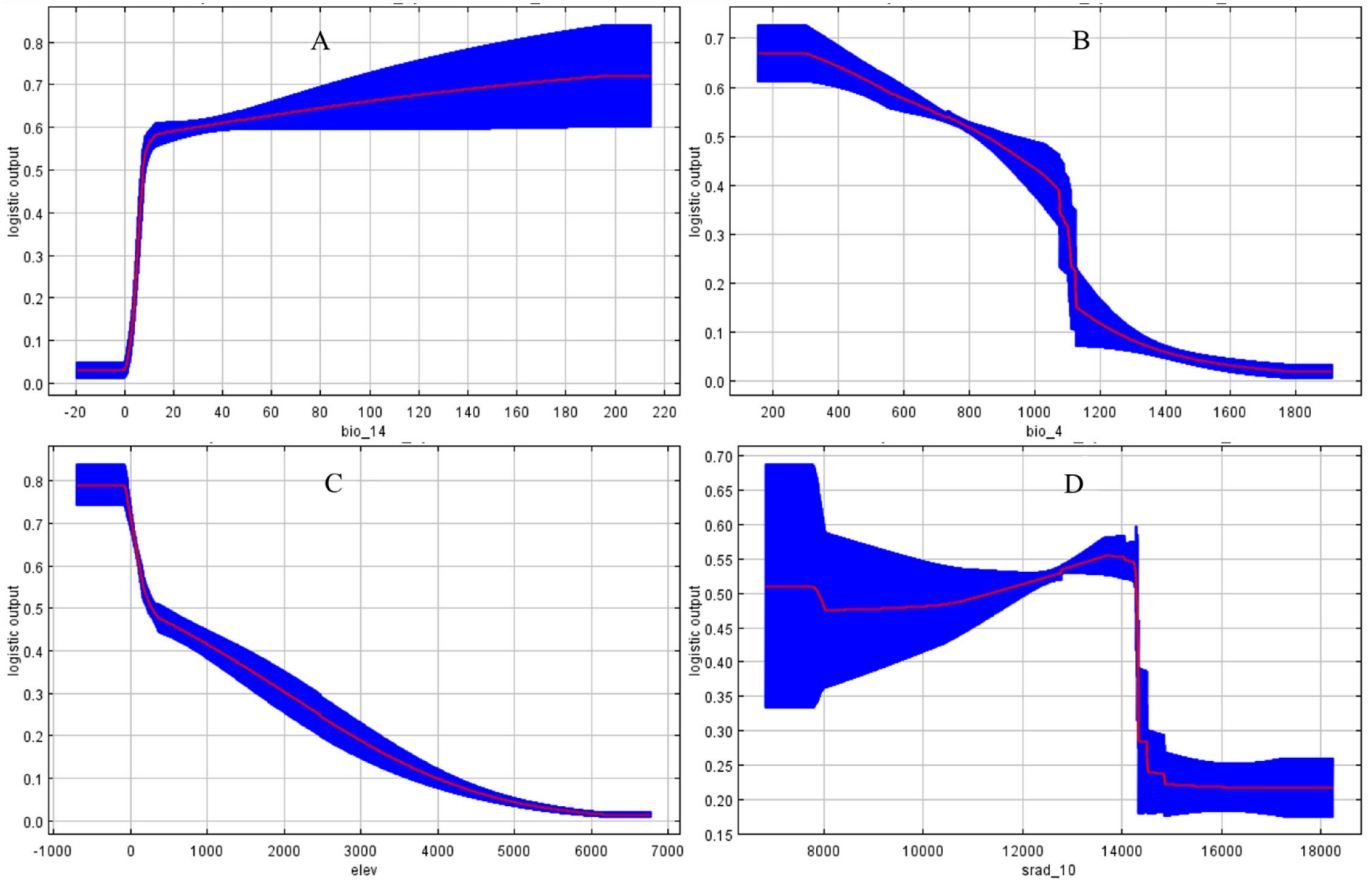
Response curves of the key environmental factor: **(A)** Precipitation of driest month, **(B)** Temperature seasonality, **(C)** Elevation, and **(D)** October solar radiation.

Based on univariate marginal effect analysis, response curves were generated for the four dominant environmental factors, as determined by their highest contribution rates. Suitable ranges and their extremes were extracted when the probability of occurrence exceeded 0.5 (Wei et al., 2023; Hou et al., 2023). Precipitation of Driest Month (Bio14) exhibited an optimal range of 7.64–214.50 mm (**Figure 7A**); Temperature Seasonality (Bio4) had an optimal range of 153.85–850.84 (**Figure 7B**); Elevation exhibited an optimal range of 0–271.65 m (**Figure 7C**); October solar radiation (Srad_10) exhibited a bimodal response pattern, with suitable ranges of 6835.50–7883.75 and 11370.31–14287.18 W/m^2^ (**Figure 7D**).

### 3.3 Current climate potential distribution of *C.* sp*eciosa*

**Figure 8:**
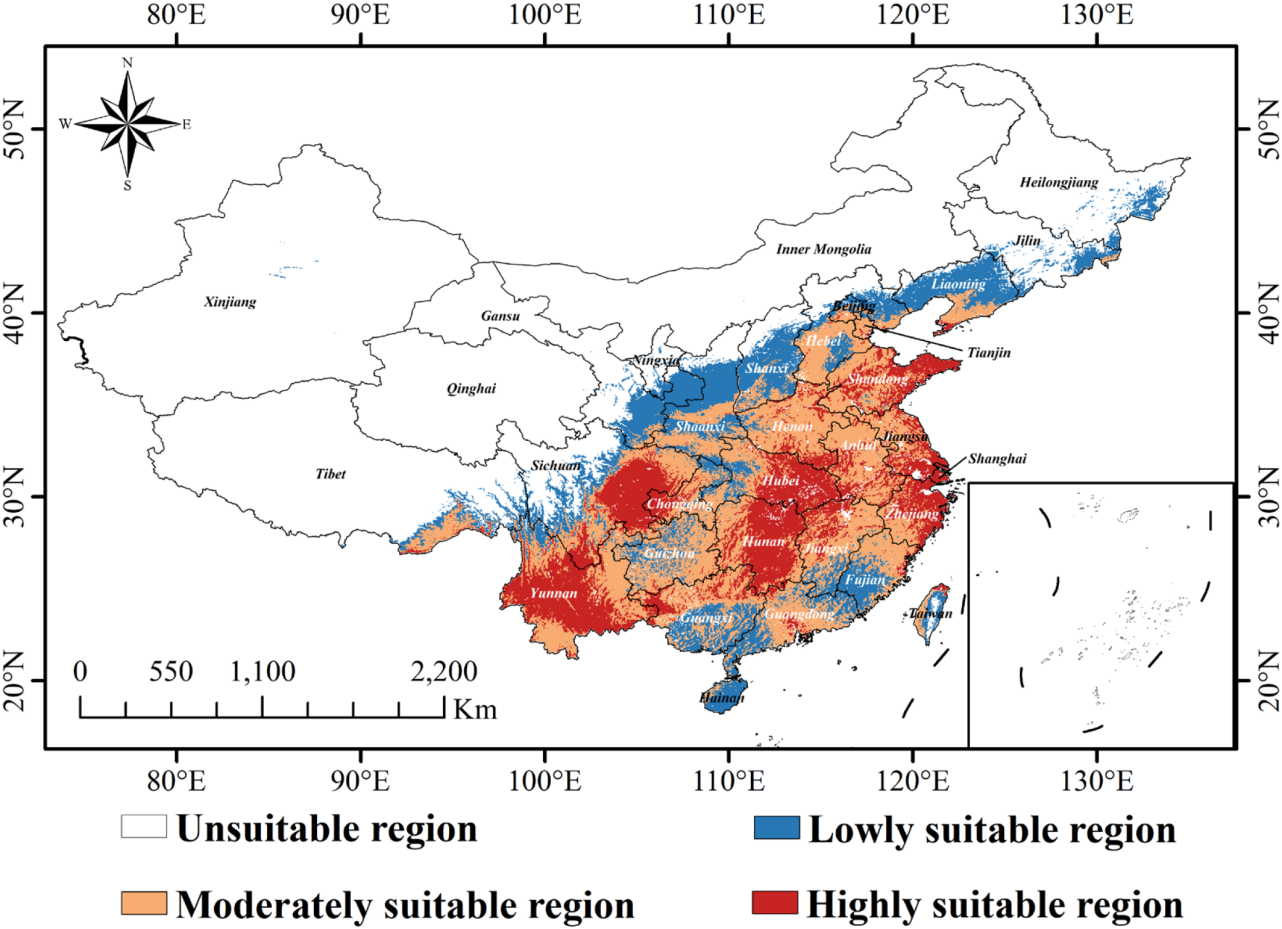
Geographic distribution of suitable habitats for *C.* sp*eciosa* under current climatic conditions in China.

According to the optimized MaxEnt model, the overall climatically suitable zone for *C.* sp*eciosa* under current climatic conditions was approximately 328.40 × 10^4^ km^2^. Within this region, the location of a highly suitable region was 88.20 × 10^4^ km^2^, accounting for 26.86% of the total suitable region. This was primarily concentrated in Shanghai, eastern Shandong, eastern Sichuan, central Yunnan, eastern Jiangsu, northeastern Zhejiang, eastern Hubei, and eastern Hunan, and formed the core region for the growth and development of the species. The moderately suitable region was the largest, reaching 148.89 × 10^4^ km^2^ and accounting for 45.34% of the total. This formed a belt surrounding the highly suitable region and extended outward, covering Anhui, Henan, Jiangxi, western Shandong, southern Hebei, western Taiwan, southeastern Guizhou, and southern Liaoning. The low-suitability region covered 91.32 × 10^4^ km^2^, accounting for 27.81% of the total region. This was primarily located at the periphery of the suitable regions and extensively distributed across Shaanxi, Shanxi, Liaoning, and southern Gansu. This distribution pattern indicates that *C.* sp*eciosa* exhibits broad ecological adaptability under the current climatic conditions, with its suitability range radiating outward from a core region in central-eastern China (**Figure 8**).

### 3.4 Distribution shifts of *C.* sp*eciosa* under future climates

The following projections for the 2050s and 2070s are based on climate data derived from a single global climate model (BCC-CSM2-MR). While this model has demonstrated good applicability for climate simulations in China (O’Neill et al., 2017), it is important to note that projections from different GCMs can vary due to differences in their inherent physical assumptions and parameterizations. Therefore, the distribution patterns and trends presented below should be interpreted as one plausible scenario under each SSP pathway, rather than a definitive forecast. The core trends are robust, but the magnitude and fine-scale spatial details may be subject to model-related uncertainty.

#### 3.4.1 Potential distribution of *C.* sp*eciosa* under future climate

Projections for the 2050s and 2070s under both SSP245 and SSP585 scenarios indicate an expansion of the total climatically suitable area for *C.* sp*eciosa* relative to the current period (**Table 2**). However, this net gain conceals a significant internal restructuring: the proportion of highly suitable habitat is projected to decrease markedly, while the proportion of low-suitability area increases across all future time slices.

**Table 2:** Climatic suitability projections: present to future (× 10^4^ km^2^).

Future climatic conditions	Decades	Lowly suitable region		Moderately suitable region		Highly suitable region		Total suitable region
		Low region	Low/total (%)	Moderately region	Moderately/total (%)	Highly region	Highly/total (%)	
—	Current	91.32	27.81	148.89	45.34	88.20	26.86	328.40
SSP2-4.5	2050S	139.45	40.04	131.56	37.78	77.25	22.18	348.26
	2070S	134.87	39.43	140.59	41.10	66.59	19.47	342.06
SSP5-8.5	2050S	128.40	37.42	139.36	40.61	75.38	21.97	343.15
	2070S	152.14	43.79	131.18	37.76	64.11	18.45	347.43

**Figure 9:**
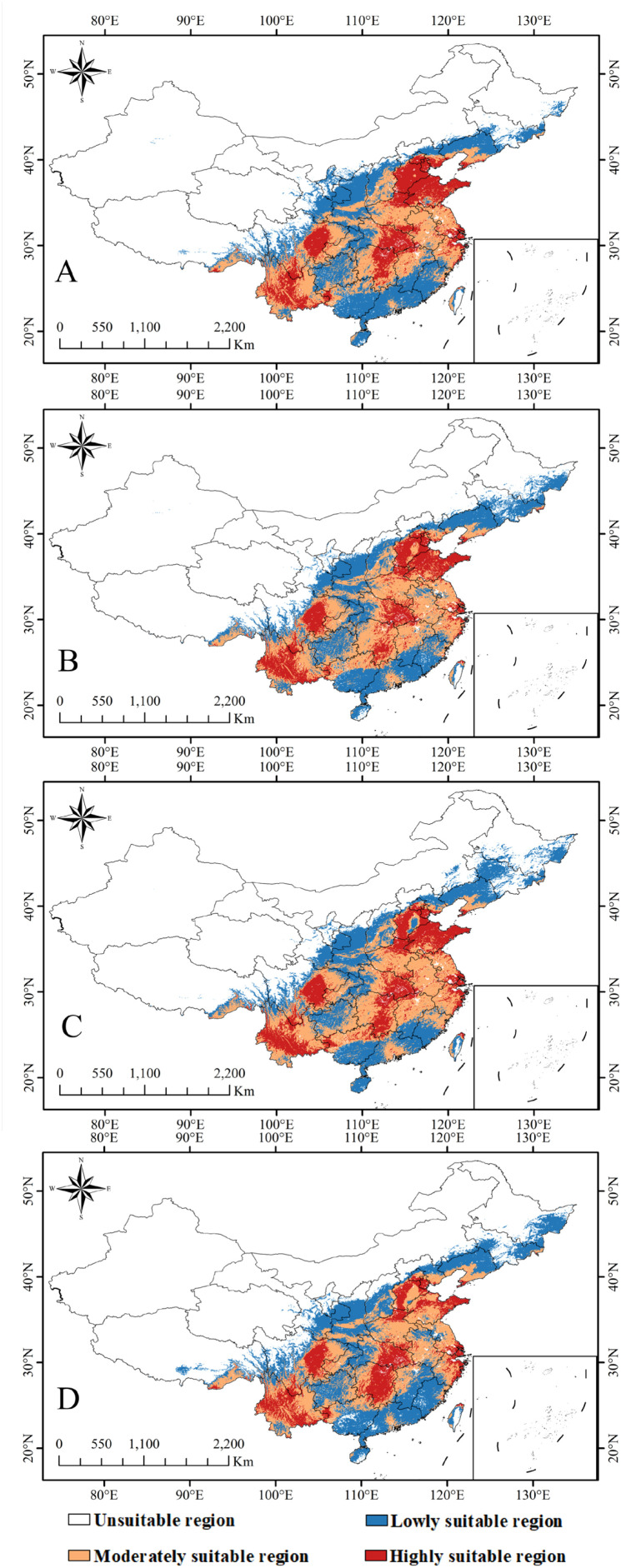
Predicted future habitat distribution of *C.* sp*eciosa*: **(A)** SSP 245, 2050s; **(B)** SSP 245, 2070s; **(C)** SSP 585, 2050s; and **(D)** SSP 585, 2070s.

Under the moderate-emission SSP245 scenario, the total suitable area reaches 348.26 × 10^4^ km^2^ in the 2050s, but the share of highly suitable area declines to 22.18%. By the 2070s, the total area decreases slightly to 342.06 × 10^4^ km^2^, with the highly suitable proportion further reduced to 19.47% (**Figures 9A, B**).

The trend of habitat quality degradation is more pronounced under the high-emission SSP585 scenario. The proportion of highly suitable area drops to 21.97% in the 2050s and to 18.45% by the 2070s, the lowest among all periods and scenarios. Concurrently, the proportion of low-suitability area increases substantially to 43.79% by the 2070s (**Figures 9C, D**).

#### 3.4.2 Comparative distribution of climatically favorable zones

Analysis of habitat gains, losses, and stability reveals a complex spatial reorganization of suitable areas for *C.* sp*eciosa* under future climates (**Supplementary Material 3**).

**Figure 10:**
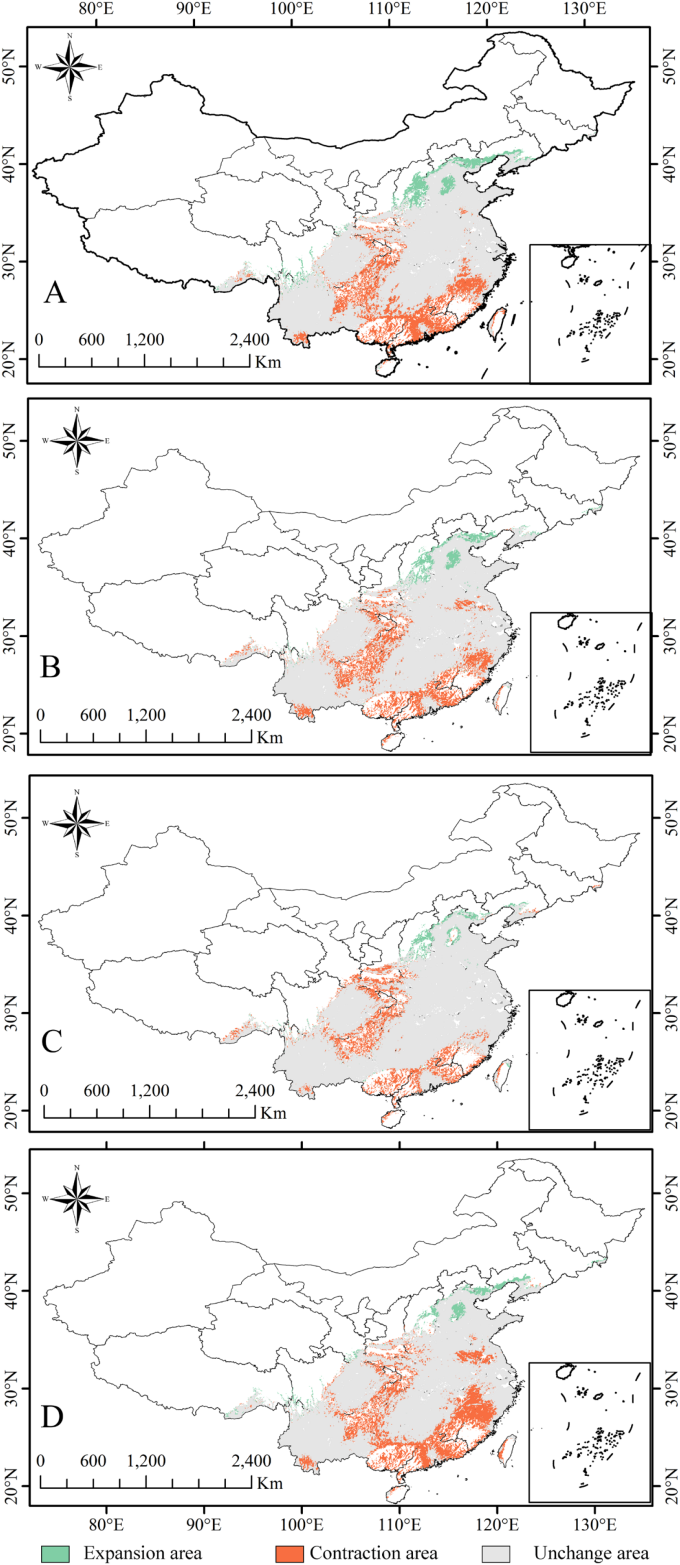
Comparative distribution of climatically favorable zones across time horizons: **(A)** SSP 245, 2050s; **(B)** SSP 245, 2070s; **(C)** SSP 585, 2050s; and **(D)** SSP 585, 2070s.

Under the SSP245 scenario, substantial spatial turnover occurs in the 2050s, with a net change of 56.04 × 10^4^ km^2^ (**Figure 10A**). By the 2070s, the process moderates; the net change decreases to 47.76 × 10^4^ km^2^, and the no-change habitat area increases, suggesting a progression toward a new spatial equilibrium under this pathway (**Figure 10B**).

In contrast, the spatial restructuring under SSP585 intensifies over time. The net change increases from 35.65 × 10^4^ km^2^ in the 2050s to 57.96 × 10^4^ km^2^ in the 2070s (**Figures 10C, D**). Concurrently, the area of stable habitat diminishes. This pattern indicates greater habitat instability and more extensive redistribution under the high-emission scenario.

The spatial dynamics are regionally heterogeneous. While the core high-suitability zone contracts, specific regions, including parts of Sichuan, Shandong, Shanxi, and Liaoning, are projected to experience an expansion of high-suitability habitat relative to current conditions. Under SSP585, newly emerging high-suitability areas are particularly notable in Liaoning, Hebei, and Shandong provinces.

### 3.5 Shift in the centroid of the suitable habitat

**Figure 11:**
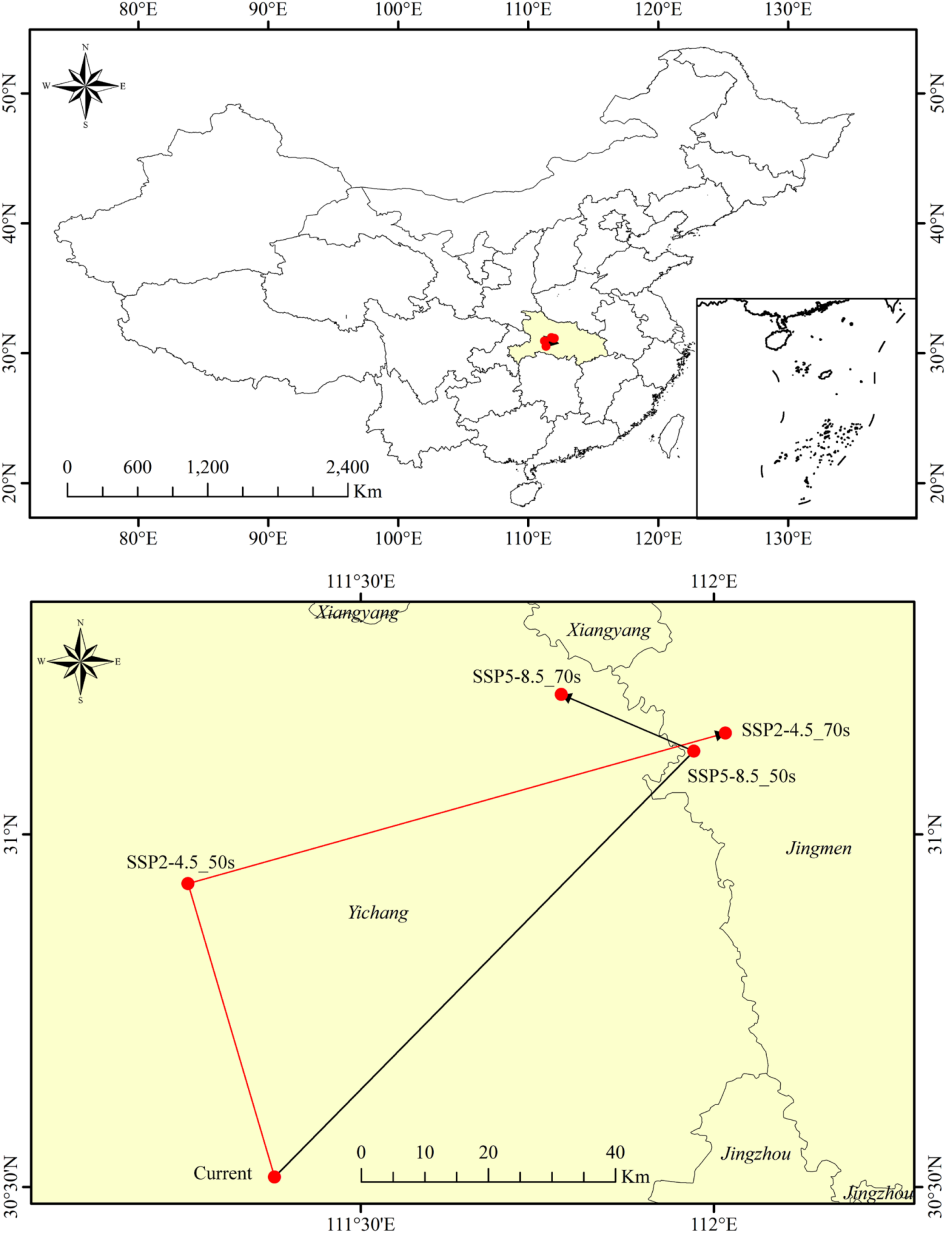
Shift in the distribution centroid of *C.* sp*eciosa* habitats under future climate scenarios.

The distribution centroid of the high-suitability area for *C.* sp*eciosa* remained within Hubei Province across all evaluated periods (**Figure 11**). Currently centered in Yidu, Yichang (111°22’41” E, 30°30’51” N), its future trajectory shows divergence: under SSP245, a northwestward shift of 47.59 km will position it in Yiling, Yichang (111°15’19” E, 30°55’49” N) by the 2050s. However, under SSP585, the suitable habitat will redistribute northeastward, leading to an 87.82 km movement of the centroid to Dongbao, Jingmen (111°58’19’’ E, 31°7’6’’ N).

From the 2050s to the 2070s, under SSP245, the center of mass will migrate 76.43 km northeastward, reaching Dongbao, Jingmen (112°0’60’’ E, 31°8’37’’ N); Under SSP585, the center of mass will shift 20.00 km northwestward, reaching Yuan’an, Yichang (111°47’2’’ E, 31°11’54’’ N) (**Supplementary Material 4**).

## 4 Discussion

### 4.1 Optimized MaxEnt model assessment

The escalating pace of climate change makes precisely projecting the geographic distribution of medicinal plants essential for informing both their conservation and sustainable management (Pecl et al., 2017). *Chaenomeles* sp*eciosa*, an important traditional Chinese medicinal herb, is experiencing increasing market demand that threatens its limited wild resources, underscoring the need for a comprehensive scientific assessment of its future suitable habitats. This study focused on *C.* sp*eciosa* by integrating geographic distribution data with critical environmental factors. The optimized model exhibits outstanding predictive capability and robustness, validating the success of the parameter tuning process. This study provides a reliable theoretical basis for scientifically formulating resource conservation strategies for *C.* sp*eciosa* and achieving precise habitat management in the context of global climate change.

### 4.2 Environmental drivers of *C.* sp*eciosa* geographic distribution

The dominant drivers were Precipitation of the Driest Month (Bio14), Temperature Seasonality (Bio4), elevation, and October Solar Radiation (Srad_10). Bio14 and Bio4 together explained approximately 66% of the distribution variance, indicating that water availability and temperature fluctuations are the primary factors shaping the distribution of *C.* sp*eciosa*. This aligns with the species’ strong adaptability to water stress, a trait suited to seasonally arid environments (Mattos et al., 2023).

Bio14 had the highest contribution rate. Its suitable range indicates that while *C.* sp*eciosa* tolerates seasonal drought, it still requires a minimum amount of water to complete its life cycle. The species possesses xeromorphic adaptations like deep roots, supporting the view that water availability is a key limiting factor for this genus (Ren et al., 2023). Bio4 was the second most influential variable. Although direct physiological evidence for *C.* sp*eciosa* is limited, studies on related temperate perennials offer insights. For example, experimental winter warming in *Ribes nigrum* delayed dormancy release, advanced budburst, and reduced fruit yield by 14–41% (Pagter et al., 2015). By analogy, pronounced temperature seasonality likely regulates dormancy, spring phenology, and reproductive success in *C.* sp*eciosa*. Furthermore, the model shows a clear preference for low-elevation habitats, consistent with its distribution in plains and hilly areas. This altitudinal restriction is common among temperate Rosaceae species to avoid climatic extremes. For instance, *Pyrus pashia* is also confined to low elevations (<1000 m), where warmer temperatures prevent frost damage during spring flowering and ensure sufficient heat for fruit maturation (Kouser et al., 2021). This shared preference highlights that avoiding the combined risks of frost and insufficient heat at high altitudes is key to the lowland distribution of several Rosaceae species, including *C.* sp*eciosa*. Srad_10 exhibits a distinct bimodal pattern. We hypothesize that these two peaks of high suitability may correspond to divergent ecological or physiological strategies. One plausible interpretation is that the lower radiation range supports carbohydrate accumulation prior to dormancy, while the higher range could be associated with enhanced synthesis of photoprotective or medicinal compounds, such as flavonoids, which are abundant in this species (Xu et al., 2023). However, this mechanistic interpretation remains speculative and stems from correlative model output combined with known phytochemical traits; it requires direct experimental validation to confirm any causal relationship between October radiation levels and specific metabolic pathways in *C.* sp*eciosa*.

### 4.3 Spatial shifts in suitable habitats of *C.* sp*eciosa*

Our projections reveal a nuanced spatial dynamic: an expansion of the total suitable area coupled with a significant contraction of high-suitability core zones and a non-linear shift of the distribution centroid within Hubei Province. This complex pattern differs from a simple poleward or upward migration often reported (Dai et al., 2022).

Although direct evidence for *C.* sp*eciosa* is currently unavailable from the literature, a study on another important Rosaceae species, *Prunus armeniaca*, under future climate scenarios similarly projected a northward expansion of its range in China, but concurrently predicted a substantial loss and fragmentation of highly suitable habitat in its traditional core cultivation regions (Ma et al., 2025). This parallel suggests that for some temperate Rosaceae species with specific climatic optima, climate change may not merely shift their range, but also degrade the quality of their habitat within historically optimal areas. Future changes in precipitation patterns are spatially more heterogeneous than uniform warming, potentially driving the complex, non-directional centroid movement observed for *C.* sp*eciosa*. The initial northeast shift under SSP585 may reflect a temporary expansion into regions like the North China Plain, where projected changes in seasonal precipitation temporarily enhance suitability, while the longer-term westward trend may indicate a tracking of more favorable future moisture regimes.

It is crucial to contextualize these projected shifts within the limitations of our modeling framework. Our reliance on a single climate model (BCC-CSM2-MR) means that the precise trajectory, distance, and regional details of the centroid migration, as well as the exact magnitude of habitat gain and loss, carry inherent uncertainty. Future studies employing multi-model ensemble approaches from CMIP6 would help quantify this uncertainty and distinguish between robust, cross-model trends and model-specific artifacts.

### 4.4 Future shift in the centroid of highly suitable regions

This study employed a center-of-mass migration analysis to reveal the spatial restructuring dynamics of *C.* sp*eciosa* habitats under future climate change scenarios. The simulation results indicated that across different climate scenarios and time periods, the distribution center of high-suitability regions for *C.* sp*eciosa* remained within Hubei. However, the migration direction and distance varied significantly. These nonlinear migration trajectories indicate that *C.* sp*eciosa*’s response to climate change is not a simple unidirectional latitudinal migration but rather a complex process influenced by the synergistic effects of multiple environmental factors (Xie et al., 2025).

However, most studies suggest that global warming drives species migration toward higher latitudes or elevations to track suitable ecological niches (Steinbauer et al., 2018; Pecl et al., 2017). The northwestward migration observed in this study may be linked to future changes in key limiting factors in central China, such as seasonal temperature (Bio4) and precipitation during the driest month (Bio14). These changes may considerably enhance suitability in certain northwestern regions, such as Shaanxi and southern Gansu. Additionally, the notable northeastward shift observed in the 2050s under SSP585 may result from the short-term enhancement of the thermo-hygric conditions under extreme emissions in regions such as the North China Plain. This is consistent with the eastward expansion of temperate tree species observed under specific scenarios in several previous studies (Fei et al., 2017; Wang et al., 2023). The shift in the direction of the center-of-mass migration revealed the time-varying effects of different climate change intensities and pathways on species distributions. Consequently, future conservation strategies should consider this spatial dynamic complexity, providing comprehensive spatiotemporal recommendations for the *ex situ* conservation and habitat management of *C.* sp*eciosa* germplasm resources.

The observed nonlinear shifts in the distribution centroid carry significant implications for conservation planning. First, the centroid’s oscillation within Hubei Province, despite the overall expansion of suitable range, indicates that the core climatic niche of *C.* sp*eciosa* is undergoing spatial restructuring rather than a simple translation. This challenges static conservation approaches focused solely on current core areas. Second, the divergent migration paths under different SSP scenarios highlight the uncertainty in predicting long-term refugia. This variability cautions against deterministic, long-range assisted migration based on short-term projections. Instead, it underscores the need for a dynamic, adaptive management strategy that prioritizes the protection of currently suitable habitats while monitoring and facilitating natural colonization into newly suitable, climatically stable regions identified over longer timeframes.

## 5 Implications for conservation and management

To ensure the sustainable use of *C.* sp*eciosa* in wild and cultivated environments, conservation strategies must integrate climate change effects and spatial dynamics. This imperative is based on simulations of its potentially suitable areas under current and future climate scenarios. Key measures include implementing habitat restoration in persistently suitable areas and establishing protected zones to prevent overexploitation. Current conservation efforts should prioritize highly suitable regions, such as eastern Hubei, eastern Hunan, eastern Sichuan, eastern Shandong, and Shanghai, with an emphasis on enhancing the protection of key habitats, implementing early monitoring, and enforcing sustainable harvesting management to enable timely responses to the risks of population decline. Promoting standardized cultivation in relatively stable and suitable areas can alleviate pressure on wild populations while meeting market demands. Furthermore, for newly emerging suitable areas under future climate scenarios, such as expanded regions in Liaoning and Hebei under SSP245 and parts of the North China Plain within the SSP585 framework, focused habitat restoration and dynamic monitoring should be conducted to facilitate natural species expansion and climate adaptation.

Critically, the projected nonlinear migration of high-suitability habitats necessitates a shift from static to proactively adaptive conservation tactics. Based on these findings, we propose an integrated strategy with three key components. First, a dynamic conservation network should be established. This network would prioritize current high-suitability zones for protection while also identifying and monitoring potential “climate corridors” that connect present and future habitats to facilitate natural dispersal. Second, any assisted migration must follow a cautious, phased approach. Translocation should target areas where habitat suitability is projected to persist and increase across multiple future periods, avoiding regions of only transient gain. Pilot trials in these areas are essential prior to large-scale implementation. Finally, ecological monitoring must be intensified along projected migration trajectories, especially within centroid shift pathways and areas of high habitat turnover (Huang et al., 2025). This enhanced monitoring will allow for the early detection of population changes, and provide a foundation for adaptive management.

## 6 Limitations of the research

First, and most pertinent to the interpretation of our future projections, is our reliance on climate data from a single model (BCC-CSM2-MR). While acknowledged for its performance in China, the use of a single GCM is a significant source of uncertainty. Different climate models exhibit systematic biases due to variations in their representation of physical processes and parameterization schemes (Zhu et al., 2022; Mamalakis et al., 2021). Consequently, the specific spatial patterns, rates of change, and absolute areas of future suitable habitat presented in this study should be viewed with appropriate caution. The overarching trends are likely indicative, but their precise quantification would benefit from an ensemble modeling approach. Furthermore, the MaxEnt model has inherent theoretical assumptions and limitations (Barthel et al., 2025). The model assumes static responses of species to environmental factors, neglecting dynamic evolutionary processes such as phenotypic plasticity or genetic adaptation. These processes enable species to adjust their ecological niches in response to future climate change. The model also assumes unrestricted migration to all newly emerging suitable habitats, whereas in reality, factors such as seed dispersal limitations and habitat fragmentation caused by human land use severely constrain the actual dispersal capacity. Finally, the model excluded biological interactions, such as pollinators, competitors, or pathogens, which are critical in determining actual species distribution boundaries (Su et al., 2022). Future research should integrate trans-boundary distribution data and employ CMIP6 multi-model ensemble averaging to quantify prediction uncertainties, thereby more comprehensively simulating distribution dynamics in a changing world.

## 7 Conclusions

This study applied an optimized MaxEnt model to project the distribution dynamics of *Chaenomeles* sp*eciosa* under climate change. The core findings reveal two critical and novel patterns: future suitable habitats will undergo internal degradation, characterized by a contraction of high-quality core areas despite a slight total range expansion; and the distribution centroid will shift in complex, nonlinear trajectories within Hubei Province, rather than following a simple directional shift.

These projections provide direct implications for conservation and agricultural planning. For *in-situ* conservation, priority must be given to stabilizing the currently highly suitable areas in central-eastern China through strengthened habitat protection and resilience-building measures. For cultivation and climate adaptation, the newly suitable regions in northern China, including Liaoning and Hebei, present strategic opportunities for trial planting and assisted migration initiatives. Implementing a dynamic monitoring system to track spatial suitability redistribution is essential for enabling adaptive management of both wild populations and cultivated resources.

The findings are subject to inherent limitations of the modeling framework, including the assumption of a static ecological niche, the exclusion of dispersal barriers and biotic interactions, and reliance on projections from a single climate model (BCC-CSM2-MR). Future research should integrate physio-ecological experiments to validate the identified climatic thresholds, employ CMIP6 multi-model ensemble approaches to quantify projection uncertainties, and incorporate data on land-use change and population genetics to better assess adaptive capacity and refine conservation strategies.

## Data Availability

The original contributions presented in the study are included in the article/**Supplementary Material**. Further inquiries can be directed to the corresponding authors.
