# Supplementary material for: Optimized MaxEnt modeling predicts the distribution change of Chaenomeles speciosa (Sweet) Nakai in China under global climate change
Source: Front Plant Sci. 2026 Feb 6;17:1737731. doi: 10.3389/fpls.2026.1737731 (PMC12920472; doi:10.3389/fpls.2026.1737731)
Supplement: Supplementary file 1 [file Table1.docx]

**Supplementary material 1.** Initial environmental factors for modelling.

| Environmental Factor | Description | Unit |
| --- | --- | --- |
| Bio1 | Annual Mean Temperature | °C |
| Bio2 | Mean Diurnal Range (Mean of monthly (max temp - min temp)) | °C |
| Bio3 | Isothermality (BIO2/BIO7) (× 100) | °C |
| Bio4 | Temperature Seasonality (Standard Deviation× 100) | °C |
| Bio5 | Max Temperature of Warmest Month | °C |
| Bio6 | Min Temperature of Coldest Month | °C |
| Bio7 | Temperature Annual Range (BIO5 - BIO6) | — |
| Bio8 | Mean Temperature of Wettest Quarter | °C |
| Bio9 | Mean Temperature of Driest Quarter | °C |
| Bio10 | Mean Temperature of Warmest Quarter | °C |
| Bio11 | Mean Temperature of Coldest Quarter | °C |
| Bio12 | Annual Precipitation | mm |
| Bio13 | Precipitation of Wettest Month | mm |
| Bio14 | Precipitation of Driest Month | mm |
| Bio15 | Precipitation Seasonality (Coefficient of Variation) | mm |
| Bio16 | Precipitation of Wettest Quarter | mm |
| Bio17 | Precipitation of Driest Quarter | mm |
| Bio18 | Precipitation of Warmest Quarter | — |
| Bio19 | Precipitation of Coldest Quarter | — |
| Elevation | Altitude | m |
| Slope | Gradient of the terrain | ° |
| Aspect | Direction the slope faces | — |
| Awc_class | Soil-effective water content | % |
| Drainage | Soil drainage class | — |
| Srad_01 | Solar radiation in January | KJ/m^2^/day |
| Srad_02 | Solar radiation in February | KJ/m^2^/day |
| Srad_03 | Solar radiation in March | KJ/m^2^/day |
| Srad_04 | Solar radiation in April | KJ/m^2^/day |
| Srad_05 | Solar radiation in May | KJ/m^2^/day |
| Srad_06 | Solar radiation in June | KJ/m^2^/day |
| Srad_07 | Solar radiation in July | KJ/m^2^/day |
| Srad_08 | Solar radiation in August | KJ/m^2^/day |
| Srad_09 | Solar radiation in September | KJ/m^2^/day |
| Srad_10 | Solar radiation in October | KJ/m^2^/day |
| Srad_11 | Solar radiation in November | KJ/m^2^/day |
| Srad_12 | Solar radiation in December | KJ/m^2^/day |
| T_gravel | Topsoil gravel content | % |
| T_ref_bulk | Topsoil reference bulk density | kg/dm^3^ |
| T_texture | Topsoil textural class | — |
| T_ph_h_2_0 | Topsoil pH | — |
| T_oc | Topsoil organic carbon content | % |
| Vapr_01 | Water vapor pressure in January | kPa |
| Vapr_02 | Water vapor pressure in February | kPa |
| Vapr_03 | Water vapor pressure in March | kPa |
| Vapr_04 | Water vapor pressure in April | kPa |
| Vapr_05 | Water vapor pressure in May | kPa |
| Vapr_06 | Water vapor pressure in June | kPa |
| Vapr_07 | Water vapor pressure in July | kPa |
| Vapr_08 | Water vapor pressure in August | kPa |
| Vapr_09 | Water vapor pressure in September | kPa |
| Vapr_10 | Water vapor pressure in October | kPa |
| Vapr_11 | Water vapor pressure in November | kPa |
| Vapr_12 | Water vapor pressure in December | kPa |
| Wind_01 | Wind speed in January | m/s |
| Wind_02 | Wind speed in February | m/s |
| Wind_03 | Wind speed in March | m/s |
| Wind_04 | Wind speed in April | m/s |
| Wind_05 | Wind speed in May | m/s |
| Wind_06 | Wind speed in June | m/s |
| Wind_07 | Wind speed in July | m/s |
| Wind_08 | Wind speed in August | m/s |
| Wind_09 | Wind speed in September | m/s |
| Wind_10 | Wind speed in October | m/s |
| Wind_11 | Wind speed in November | m/s |
| Wind_12 | Wind speed in December | m/s |

**Supplementary material 2.** Variable significance of optimized environmental predictors.

| Environmental Factor | Percent contribution | Permutation importance |
| --- | --- | --- |
| Bio_14 | 39.1 | 3.9 |
| Bio_4 | 27 | 36.9 |
| Elevation | 13.7 | 36.2 |
| Srad_10 | 6.5 | 5 |
| Srad_08 | 4.4 | 6.1 |
| Awc_class | 3.6 | 1.5 |
| Bio_15 | 2.1 | 3.1 |
| Aspect | 1.5 | 1.3 |
| Bio_3 | 1.4 | 4 |
| Slope | 0.9 | 1.9 |

**Supplementary material 3.** Change rates of suitable regions in different periods (× 10^4^ km^2^).

| Future climatic conditions | Decades | Expansion | Contraction | Unchange | Total region change |
| --- | --- | --- | --- | --- | --- |
| Current_SSP2-4.5 | 2050S | 13.62 | 42.42 | 197.05 | 56.04 |
|  | 2070S | 8.93 | 38.83 | 200.55 | 47.76 |
| Current_SSP5-8.5 | 2050S | 6.24 | 29.41 | 210.23 | 35.65 |
|  | 2070S | 7.85 | 50.11 | 189.26 | 57.96 |

Total region change = Expansion + Contraction

**Supplementary material 4.** Spatial trajectory of the range core in *C. speciosa* across time periods.

| Shared socioeconomic pathway | Time | Center of mass migration direction | Centroid migration distance/km | Elevation change | Elevation change distance/m |
| --- | --- | --- | --- | --- | --- |
| Current_SSP2-4.5 | 2050s | NW | 47.59 | Increase | 404 |
|  | 2070s | NE | 76.43 | Decrease | 215 |
| Current_SSP5-8.5 | 2050s | NE | 87.82 | Increase | 91 |
|  | 2070s | NW | 20.00 | Increase | 301 |

Notes: NW = Northwest; NE = Northeast.
